# Network analysis of *KLF5* targets showing the potential oncogenic role of *SNHG12* in colorectal cancer

**DOI:** 10.1186/s12935-020-01527-x

**Published:** 2020-09-07

**Authors:** Qi Liao, Linbo Chen, Ning Zhang, Yang Xi, Shiyun Hu, Derry Minyao Ng, Fatma Yislam Hadi Ahmed, Guofang Zhao, Xiaoxiang Fan, Yangyang Xie, Xiaoyu Dai, Yanping Jin, Jiaxin Ge, Changzheng Dong, Xinjun Zhang, Junming Guo

**Affiliations:** 1 Department of Preventative Medicine, Zhejiang Provincial Key Laboratory of Pathophysiology, Ningbo University School of Medicine, Ningbo, 315211 Zhejiang China; 2 The Affiliated Hospital of School of Medicine, Ningbo University, Ningbo, 315020 China; 3 Department of Biochemistry and Molecular Biology, Zhejiang Provincial Key Laboratory of Pathophysiology, Ningbo University School of Medicine, Ningbo, 315211 Zhejiang China; 4 Department of Gastroenterology, The Affiliated People’s Hospital of Ningbo University, Ningbo, 315040 Zhejiang China; 5 Hua Mei Hospital, University of Chinese Academy of Science, Ningbo, 315000 China; 6 Department of Gastrointestinal Surgery, The First Affiliated Hospital, Sun Yat-Sen University, Guangzhou, 510080 China

**Keywords:** Long non-coding RNA (lncRNA), *KLF5*, *SNHG12*, Colorectal cancer, Bioinformatics

## Abstract

**Background:**

*KLF5* is a member of the Kruppel-like factor, subfamily of zinc finger proteins that are involved in cancers. *KLF5* functions as a transcription factor and regulates the diverse protein-coding genes (PCGs) in colorectal cancer (CRC). However, the long non-coding RNAs (lncRNAs) regulated by *KLF5* in CRC are currently unknown.

**Methods:**

In this study, we first designed a computational pipeline to determine the PCG and lncRNA targets of *KLF5* in CRC. Then we analyzed the motif pattern of the binding regions for the lncRNA targets. The regulatory co-factors of *KLF5* were then searched for through bioinformatics analysis. We also constructed a regulatory network for *KLF5* and annotated its functions. Finally, one of the *KLF5* lncRNA targets, *SNHG12*, was selected to further explore its expression pattern and functions in CRC.

**Results:**

We were able to identify 19 lncRNA targets of *KLF5* and found that the motifs of the lncRNA binding sites were GC-enriched. Next, we pinpointed the transcription factors *AR* and *HSF1* as the regulatory co-factors of *KLF5* through bioinformatics analysis. Then, through the analysis of the regulatory network, we found that *KLF5* may be involved in DNA replication, DNA repair, and the cell cycle. Furthermore, in the cell cycle module, the *SNHG12* up-regulating expression pattern was verified in the CRC cell lines and tissues, associating it to CRC invasion and distal metastasis. This indicates that *SNHG12* may play a critical part in CRC tumorigenesis and progression. Additionally, expression of *SNHG12* was found to be down-regulated in CRC cell lines when *KLF5* expression was knocked-down by siRNA; and a strong correlation was observed between the expression levels of *SNHG12* and *KLF5*, further alluding to their regulatory relationship.

**Conclusions:**

In conclusion, the network analysis of *KLF5* targets indicates that *SNHG12* may be a significant lncRNA in CRC.

## Background

Colorectal cancer (CRC) is one of the most common cancers in digestive system. CRC is the third most frequent malignancy in males and the second most frequent in females worldwide [1]. Approximately 1.4 million new cases of CRC and 700,000 CRC-related deaths are reported worldwide each year [1], suggesting that it is a major public health problem. Unfortunately, treatments such as aggressive surgical management and chemotherapy have a limited overall impact on cure rates and long-term survival rates, partially due to the complicated pathogenesis and underlying regulatory mechanisms of CRC [2].

Kruppel-like factor 5 (*KLF5*) is one of the 17 known members of the Kruppel-like factor subfamily of zinc finger proteins [3]. Increasing evidences have revealed that *KLF5* is involved in various cancers, such as prostate cancer [4], non-small-cell lung cancer [5], and bladder cancer [6], as well as other diseases [7, 8]. Interestingly, *KLF5* is critical for intestinal development and homeostasis. Furthermore, it has been detected to have a positive role in intestinal tumorigenesis [9]. *KLF5* can also activate beta-catenin with lysophosphatidic acid to stimulate the proliferation of colon cancer cells [10]. *KLF5* also participates in the cell cycle, inducing the expression of several cell cycle-related genes including cyclin D1 and cyclin B [11, 12]. Owing to the important role of *KLF5* in CRC, a number of studies have aimed to identify novel small molecule compounds that can inhibit the function of *KLF5*, yielding potential therapeutic targets for the treatment of CRC [13, 14]. For example, ML264, a novel small molecule compound, was able to inhibit the proliferation of CRC in vitro by suppressing the expression of *KLF5* [14].

Long non-coding RNAs (lncRNAs) are transcripts that consist of more than 200 nucleotides and have limited to no protein-coding capacity [15]. lncRNAs are considered to be a relatively new type of regulatory RNAs. Emerging evidences have shown that lncRNAs have prominent roles in human diseases, owing to its participation in a large range of biological processes [16, 17] as well as in cancer development [18, 19]. Some lncRNAs are up-regulated in cancers and act as oncogenes, such as *LINC00941* in gastric cancer [20], HOX transcript antisense RNA (*HOTAIR*) in hepatocellular carcinoma and CRC [21, 22], and *H19* in bladder and prostate cancer [23, 24]. Other lncRNAs, including Growth Arrest Specific 5 (*GAS5*), Maternally Expressed Gene 3 (*MEG3*), and *LOC285194* have been reported to function as tumor suppressors in cancers [25–27]. *KLF5* has been shown to be regulated by *CASC15* and *PVT1* in breast cancer [28, 29] along with *MALAT1* in pulmonary artery hypertension [30]. However, the role of lncRNAs in CRC remains unknown.

As previous reports, we developed several bioinformatics tools [31, 32] and constructed a comprehensive regulatory network of lncRNAs in CRC [2]. Acknowledging the critical role of *KLF5* and lncRNAs, we hypothesize that *KLF5* may regulate several specific pivotal lncRNAs in CRC [33]. In this research, we initially designed a computational pipeline to investigate the lncRNA and protein-coding gene (PCG) targets of *KLF5* in CRC. Next, we analyzed the DNA motif pattern of the binding regions on the lncRNA targets and predicted for the regulatory co-factors associated with *KLF5*. Furthermore, we established a *KLF5* regulatory network based on co-expression relationships and protein–protein interactions (PPIs) to identify the functional modules related to *KLF5*. Finally, one of the lncRNA targets, termed *SNHG12*, which has been shown to be increased in osteosarcoma tissues in contrast with normal tissue [34], was selected to explore its expression patterns in CRC tumors and cell lines, potential functions, and association with clinical–pathological factors of patients with CRC. In addition, expression of *SNHG12* was observed after knocking-down expression of *KLF5* in CRC cell line with siRNA and the relationship between the expression levels of *SNHG12* and *KLF5* in CRC tissues or adjacent non-tumorous tissues was reviewed to verify their regulatory relationship.

## Materials and methods

### Identification of CRC associated lncRNAs and PCGs

The expression datasets for lncRNAs and PCGs in primary cancer, normal colon tissue, and metastasized CRC were collected from the GEO database (GSE50760) [35]. Differential expression analysis was performed between the normal colon and primary cancer, as well as normal colon and metastasized cancer, respectively, using a *t*-test with False Discovery Rate (FDR)-adjusted *p*-value less than 0.05. To obtain more accurate results, the differentially expressed genes were obtained based on the intersection with the Cancer RNA sequencing (RNA-Seq) Nexus database [36], which includes expression profiles from The Cancer Genome Atlas (TCGA).

### The potential PCG and lncRNA targets of *KLF5* in CRC

First, using the Cistrome database (https://cistrome.org/) [37], we collected the target genes identified from the chromatin immunoprecipitation DNA sequencing (ChIP-Seq) datasets of *KLF5* in two colon adenocarcinoma cell lines: the GP5d cell line with transfecting and non-transfecting RAD21 short interfering RNA (GSM1240834, GSM1240820) [38], as well as the LoVo cell line, which was blocked by double thymidine to cause cell cycle arrest in the early S phase, was cultured in a medium containing nocodazole in M-phase synchronization (GSM1208642, GSM1242268, GSM1242274) [38]. Overall, five samples of *KLF5* ChIP-Seq datasets were obtained, plus PCGs and lncRNAs with a *KLF5* binding site in at least two samples were selected for the first step. Second, the differently expressed lncRNAs and PCGs were obtained from the intersection results of the RNA-Seq dataset (GSE50760) and Cancer RNA-Seq Nexus database. Third, the potential lncRNA and PCG targets of *KLF5* were picked up from the intersection of the results obtained in the first and second steps. Finally, the lncRNA targets of *KLF5* with higher confidence were further selected based on the lncRNA PCG co-expression relationships.

### Motif analysis of *KLF5* binding sites

We downloaded the *KLF5* binding peaks of ChIP-seq datasets from the Cistrome database [37], and then mapped them to the genome regions of lncRNA and PCG targets in GENCODE v25 database [39] with 2 kb extended at the transcription start site. Furthermore, MEME software was used to analyze the motif pattern of *KLF5* binding sites [40, 41].

### Prediction of *KLF5* regulatory co-factors in CRC

We first downloaded the ChIP-Seq profiles of all other transcription factors (TFs) from the Cistrome database [37] and obtained the target genes of the TFs detected from each ChIP-Seq profile. Then we proposed a two-step enrichment analysis method to identify the TFs that may co-regulate with *KLF5* (Additional file 1: Figure S1).

In the first step, we calculated the significance of enrichment by overlapping, between the *KLF5’*s targets and the target genes of each ChIP-Seq profile collected, using a hypergeometric test. Then the ChIP-Seq profiles with *p*-values less than 0.01 ranked in the top 50 were selected in a descending order, denoted as “sigChIP-Seq-profiles”. The formula of the hypergeometric test was as follows:

$${\varvec{p}}\text{-value}= \sum_{{\mathrm{N}}_{\mathrm{s}}}^{\mathrm{min}\left({\mathrm{N}}_{\mathrm{c}},{\mathrm{N}}_{\mathrm{k}}\right)}\frac{\left(\begin{array}{c}{\mathrm{N}}_{\mathrm{k}}\\ {\mathrm{N}}_{\mathrm{s}}\end{array}\right)\left(\begin{array}{c}{{\mathrm{N}}_{\mathrm{T}}-\mathrm{N}}_{\mathrm{k}}\\ {\mathrm{N}}_{\mathrm{c}}- {\mathrm{N}}_{\mathrm{s}}\end{array}\right)}{\left(\begin{array}{c}{\mathrm{N}}_{\mathrm{T}}\\ {\mathrm{N}}_{\mathrm{c}}\end{array}\right)},$$

where N_T_ represents the total number of all genes (lncRNAs or PCGs) obtained from the GENCODE database [42]; N_c_ represents the number of other TFs’ targets from each ChIP-Seq profile; N_k_ represents the number of *KLF5*’s targets; and N_s_ represents the number of common targets between N_k_ and N_c_.

In the second step, for each TF involved in the dataset of “sigChIP-Seq-profiles” constructed above, we compared the number of ChIP-Seq profiles for this TF in this dataset and the pool of all ChIP-Seq profiles, using the following hypergeometric test:

$${\varvec{p}}\text{-value}= \sum_{{\mathrm{N}}_{\mathrm{s}}}^{\mathrm{min}\left({\mathrm{N}}_{\mathrm{e}},{\mathrm{N}}_{\mathrm{tf}}\right)}\frac{\left(\begin{array}{c}{\mathrm{N}}_{\mathrm{tf}}\\ {\mathrm{N}}_{\mathrm{s}}\end{array}\right)\left(\begin{array}{c}{{\mathrm{N}}_{\mathrm{T}}-\mathrm{N}}_{\mathrm{tf}}\\ {\mathrm{N}}_{\mathrm{e}}- {\mathrm{N}}_{\mathrm{s}}\end{array}\right)}{\left(\begin{array}{c}{\mathrm{N}}_{\mathrm{T}}\\ {\mathrm{N}}_{\mathrm{e}}\end{array}\right)},$$

where N_T_ represents the total number of ChIP-Seq profiles downloaded from the Cistrome database [37]; N_e_ represents the number of “sigChIP-Seq-profiles” selected in the first step (that is, 50 or less); N_tf_ represents the number of ChIP-Seq profiles corresponding to the same TF in the pool of all ChIP-Seq profiles; N_s_ represents the number of significant ChIP-Seq profiles corresponding to the same TF in “sigChIP-Seq-profiles”. Finally, TFs with *p*-values less than 0.01 and the number of significant ChIP-Seq profiles not less than 3 were determined to be the co-regulatory TFs of *KLF5*.

### *KLF5* regulation network construction and module analysis

We constructed a *KLF5* regulatory network by combining the targets of *KLF5*, the co-expression relationships in CRC among *KLF5* with its lncRNA and PCG targets, as well as the PPIs among *KLF5* and protein-coding targets. Next, we downloaded three datasets from the Co-LncRNA database, obtaining the co-expression relationships in CRC based on Spearman correlation [43], the final co-expression links consisted of relationships observed in at least two datasets along with the PPIs from the HPRD database [44]. Additionally, we used the MCL algorithm with default parameters to identify the modules [45]. Then, we obtained calculations of the enriched gene ontology biological process using the hypergeometric test with FDR-adjusted *p-*values less than 0.05.

### Function prediction of *SNHG12* based on co-expression network

The co-expressed PCG partners of *SNHG12* were obtained in the same way as stated above. We organized a lncRNA PCG co-expression network for CRC based on three CRC datasets obtained from the Co-LncRNA database [43], requiring that the co-expressed links should be observed in at least two datasets.

### Patients and tissue samples

We obtained CRC tissues and their paired adjacent-normal tissues between the years 2012 to 2015 from surgical patients at the Affiliated Hospital of Ningbo University School of Medicine and the First Affiliated Hospital of Sun Yat-sen University, China. All tissue specimens were immediately placed in an RNA-fixer Reagent (Bioteke, Beijing, China) after surgical resections and then stored at − 80℃ until use. The diagnoses of all 111 CRC patients were based on histopathological evaluation using the clinical staging performed according to the 7th edition of the American Joint Committee on Cancer (AJCC) Cancer Staging Manual. It is important to mention that no patients received any treatment before surgery and all patients were anonymous with written informed consent and that all aspects of this study were approved by the Human Research Ethics Committee of Ningbo University with the methods performed in accordance with the relevant guidelines and regulations.

### RNA extraction, reverse transcription, and qRT-PCR detection

According to the manufacturer’s protocol, TRIzol reagent (Ambion, Carlsbad, CA, USA) was used to extract total RNA from tissues and cultured cells. We reverse-transcribed 2 μg total RNA into cDNA using the PromegaGoScript Reverse Transcription System (Promega, Madison, WI, USA). The *SNHG12* expression level was normalized to β-actin. Furthermore, qRT-PCR was performed using GoTaq qPCR master mix (Promega) on the Mx3005P QPCR System (Stratagene, La Jolla, CA, USA), the 25 μl PCR reaction mix contained 12.5 μl GoTaq qPCR Master Mix, 5 μl cDNA product, 1 μl forward primer, 1 μl reverse primer, and 5.5 μl nuclease-free water. Overall, a total of 45 cycles of amplification were performed after 10 min of preheating. Each cycle consisting of 95 °C for 15 s, 60 °C for 30 s, and 72 °C for 30 s. The primer sequences were as follow: 5′-TCTGGTGATCGAGGACTTCC-3′ (forward) and 5′-ACCTCCTCAGTATCACACACT-3′ (reverse) for *SNHG12*, 5′-CCTGGTCCAGACAAGATGTGA-3′ (forward) and 5′-GAACTGGTCTACGACTGAGGC-3′ (reverse) for *KLF5* and 5′-CTCCTTAATGTCACGCACGAT-3′ for β-actin. The expression levels of *SNHG12* were calculated using the Δ*C*_t_ method and relative expression levels of *KLF5* and *SHNG12* were calculated using the 2^−ΔΔ*C*t^ method [46]. Each experiment was performed in triplicate; and all results were expressed as the means ± SD.

### Cell culture

The human colon epithelial cell line (NCM460) and the four CRC cell lines (HCT116, HT29, SW620 and COLO-205) were purchased from the Chinese Academy of Sciences Cell Bank (Shanghai, China). The cells were maintained by being supplemented with 10% fetal bovine serum (FBS) and grown in humidified air containing 5% CO_2_ at 37 °C in RPMI 1640 or McCoy's 5A (Invitrogen, USA) medium.

### siRNA and transfection

The Lipofectamine^®^ RNAiMAX Transfection Reagent (Invitrogen, Germany) was used for the knock-down of *KLF5* gene following the manufacturer’s instructions. The sequence of the *KLF5* siRNA (siKLF5) was: 5′‐AAAGTATAGACGAGACAGTGC‐3′, which was collected from a previous study [47]. Besides, the negative control siRNA (siNC) was: 5′-CUUACGCUGAGUACUUCGATT-3′. The siRNAs were synthesized by Genechem company (Shanghai, China). The siRNA knock-down experiment was performed for 72 h, then proteins were extracted.

### Western blot analysis

Total proteins were extracted and separated by SDS-PAGE. Primary antibodies against KLF5 (#DF7135, Affinity, Cincinnati, OH, USA) and GAPDH (#AP0063, Bioworld Antibodies, Bloomington, MN, USA) were used.

### Statistical analysis

All statistical analyses were performed using SPSS version 13.0 (SPSS, Chicago, IL, USA), R and GraphPad Prism 6.0 (GraphPad Software, La Jolla, CA, USA). The expression differences between the CRC tissues and adjacent non-cancer tissues were evaluated using paired sample *t*-tests. We also used the rank-sum test to evaluate the expression between the CRC cell lines and NCM460. Furthermore, independent sample *t*-tests and One-way Analysis of Variance (ANOVA) test was assisted in analyzing the correlation between the expression levels and clinical–pathological characteristics of CRC patients and the comparison of expression between siNC and siKLF5 group. To further illustrate the diagnostic values, we used the Receiver Operating Characteristic (ROC) curve for assessment. A two-sided *p*-value < 0.05 was considered to indicate statistical significance.

## Results

### Identification of PCG and lncRNA targets of *KLF5* in CRC

**Fig. 1:**
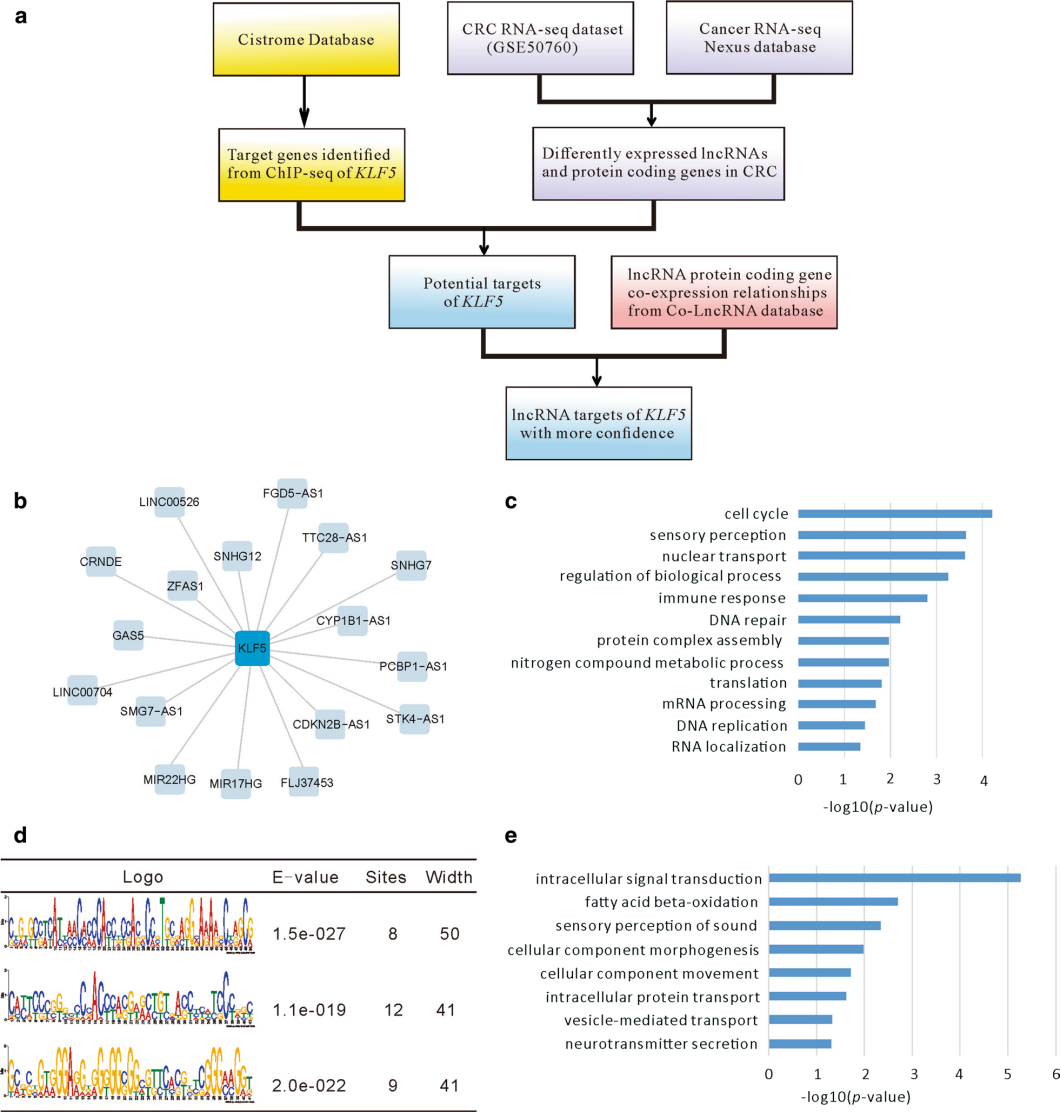
Identification of lncRNA targets of *KLF5* in CRC. **a** This represents the computational pipeline for identifying the lncRNA targets of *KLF5* in CRC. **b** The visualization of the *KLF5* lncRNA target network. The central node is *KLF5*. Other nodes are lncRNA targets of *KLF5*. **c** The enriched GO BP terms of the *KLF5* targets that were up-regulated in CRC demonstrated that its major functions are involved with cell cycle, DNA repair, and DNA replication. **d** The three motif patterns of the *KLF5* binding sites on the lncRNAs (with most occurrences) are presented in MEME software. **e** The enriched GO BP terms of the *KLF5* targets that were down-regulated in CRC showing that the functions are related to signal transduction

We designed a computational pipeline to obtain the PCG and lncRNA targets of *KLF5* in CRC (Fig. 1a). First, we collected the differently expressed PCGs and lncRNAs based on the intersection between the CRC RNA-Seq dataset GSE50760 [35] and the cancer RNA-Seq Nexus database [36]. Among these differentially expressed genes, 71 lncRNAs and 3,464 PCGs were down-regulated in CRC, while 73 lncRNAs and 3786 PCGs were up-regulated. Some of the lncRNAs, such as *UCA1*, *H19*, *GAS5*, and *PVT1*, have previously been reported to be associated with CRC [48–51]. Second, we required that the lncRNA targets of *KLF5* should have the *KLF5* binding sites at the corresponding promoter regions (from − 2000 to 200 bp of TSS), based on ChIP-Seq datasets for *KLF5* [38]. As a result, 19 lncRNA and 1744 PCG targets were identified, among which 10 lncRNAs and 956 PCGs were up-regulated while 9 lncRNAs and 788 PCGs were down-regulated in CRC, respectively (Fig. 1b, Additional file 2: Table S1 and Additional file 3: Table S2).

Function enrichment analysis established that the up-regulated targets of *KLF5* in CRC were correlated with cell cycle, immune response, DNA repair, and DNA replication, while the down-regulated targets were linked to intracellular signal transduction, fatty acid beta-oxidation, and sensory perception of sound (Fig. 1c, e). Several lncRNAs, such as *GAS5*, have shown to be involved in CRC development and progression, reflecting the accuracy of our analysis. *KLF5* may also be able to regulate key lncRNAs in CRC. Specifically, 11 lncRNAs, including *SNHG12*, were co-expressed with *KLF5* in CRC. This finding enhanced our confidence that there are probable lncRNA targets from our established coding non-coding gene co-expression network [32] (Additional file 2: Table S1).

### Motif analysis of *KLF5* binding sites on lncRNA targets

*KLF5* is considered to be a GC-box binding factor [52]. According to the transcription assays in vitro, *KLF5* level of enhanced transcription depends on the presence of GC boxes in the promoter region of the target gene [53]. However, this conclusion was based on PCGs. We further investigated the binding patterns of *KLF5* on lncRNA targets by analyzing the ChIP-Seq datasets from the Cistrome database and identified 62 and 4180 peaks for lncRNA and PCG targets of *KLF5*, respectively [37]. Although there were far fewer peaks for lncRNAs than for PCGs, the lengths (mean = 258 bp and 275 bp, respectively, *p* > 0.05) and the signal levels (mean = 12.9 and 13.7, respectively, *p* > 0.05) were similar in both cases. Furthermore, MEME software was used to demonstrate the motif patterns of the peaks for lncRNAs, resulting in the detection of GC sequence-enriched motifs, suggesting the presence of active transcriptional regulation [54] (Fig. 1d). In a previous study, *KLF5* was also found to regulate target genes with a GC-rich motif in a sequence-specific manner in lymphoid cells and tissues [55], indicating that the transcription regulation mechanism of *KLF5* to lncRNAs is identical to that of PCGs.

### Identification of regulatory co-factors for *KLF5*

Some TFs have been shown to co-operate with other TFs to regulate the expression of downstream genes. In a bioinformatics analysis, *KLF5* was discovered to interact with several TFs including *CREB1*, *Sp1*, *MYC*, *ER*, and *AR* to regulate gene expression [56]. To illustrate the regulatory mechanism of *KLF5* in CRC, we obtained the target genes of other TFs based on 5500 ChIP-Seq profiles from the Cistrome database [37] and proposed a two-step enrichment analysis method to identify candidate TFs that may co-operate with *KLF5* (Additional file 1: Figure S1). First, we analyzed the enrichment values of the overlapping genes between the lncRNA or PCG targets of *KLF5* and each target set detected from 5500 ChIP-Seq profiles of other TFs in diverse cell types, using a hypergeometric test. Second, according to the results of the first step, we can select the significant ChIP-Seq profiles with a *p*-value less than 0.01 and ranked the top 50 in descending order, denoted as “sigChIP-Seq-profiles”. Third, for each TF, we calculated the significance of the proportion of this TF involved in the “sigChIP-Seq-profiles” compared with the background, using the same hypergeometric test. Finally, the TFs with a number of significant ChIP-Seq profiles not less than 3 and a *p*-value less than 0.01 were determined to be the regulatory co-factors.

**Fig. 2:**
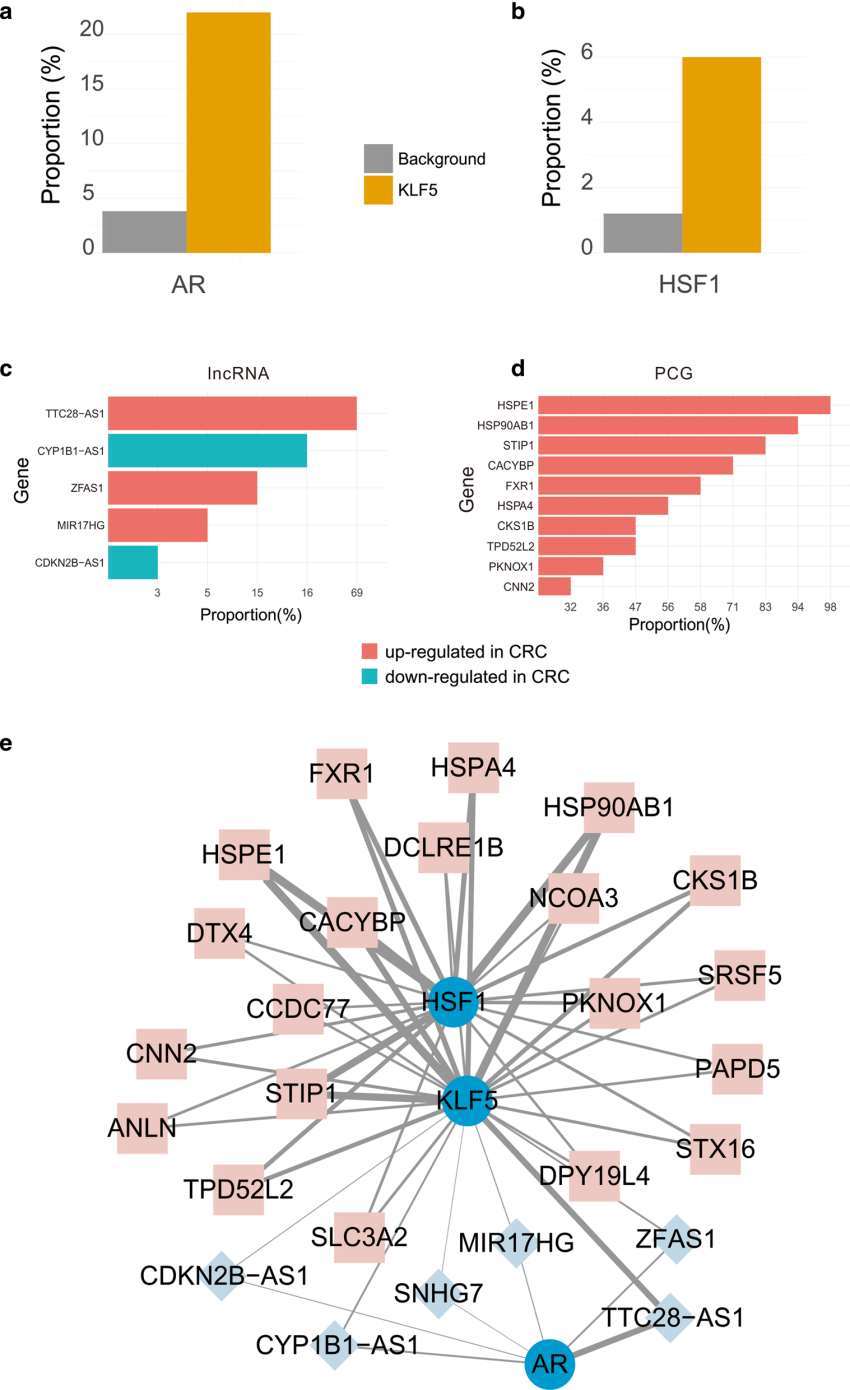
Regulatory co-factors of *KLF5* in CRC. **a**, **b** The orange bar represents the proportion of the ChIP-Seq profiles of *AR* (**a**) and *HSF1* (**b**) whose targets were enriched with *KLF5*’s, while the grey bar represents the proportion at random. **c** Common lncRNA targets of *KLF5* and *AR*. The bar represents the percentage of the gene that was detected by ChIP-Seq profiles corresponding to AR. The red bar represents the up-regulated genes in CRC while the green bar represents the down-regulated genes in CRC. **d** The common PCG targets of *KLF5* and *HSF1*. The bar represents the percentage of the gene that was detected in the ChIP-Seq profiles corresponding to *HSF*. The red bar represents the up-regulated genes in CRC. The picture shows the top 10 PCGs. **e** Network visualization of the co-regulatory network for *KLF5* in CRC. The squares nodes represents the PCG, the circle nodes represents TF while the diamond nodes represents lncRNA. Furthermore, the width of the edge represents the percentage of ChIP-Seq profiles that detected the gene as the target of the corresponding TF (*AR* or *HSF1*)

Consequently, we found that the *AR* was a co-regulatory TF of *KLF5* with respect to the lncRNA targets (Fig. 2a). In an initial study, *KLF5* was revealed to be a target of *AR* and to promote cancer progression in prostate cancer [57]. *AR* was also predicted to be a co-factor of *KLF5* in a bioinformatics analysis [56]. These results suggest the presence of a relationship between *AR* and *KLF5*. Additionally, *HSF1* was established to be a co-regulator TF of *KLF5* for the PCG target (Fig. 2b).

Thus, we explored the common targets of *KLF5* and its co-regulatory TFs. For *AR*, the same lncRNA targets are *TTC28-AS1*, *CYP1B1-AS1*, *ZFAS1*, *MIR17HG*, *CDKN2B-AS1*, and *SNHG7* (Fig. 2c), whereas the common PCG targets of *HSF1 and KLF5* included *HSPE1*, *HSP90AB1*, *STIP1*, *CACYBP*, *FXR1*, and *CKS1B* (Fig. 2d). A visualization of the network for *KLF5* and its co-regulatory TFs is presented in Fig. 2e. Some of these targets have been confirmed to be associated with CRC such as *MIR17HG* [58] and *SNHG7* [59]. *KLF5* may co-operate or form a transcription complex with *AR* or *HSF*1 to promote tumorigenesis in CRC.

### Module analysis of *KLF5* regulatory network

**Fig. 3:**
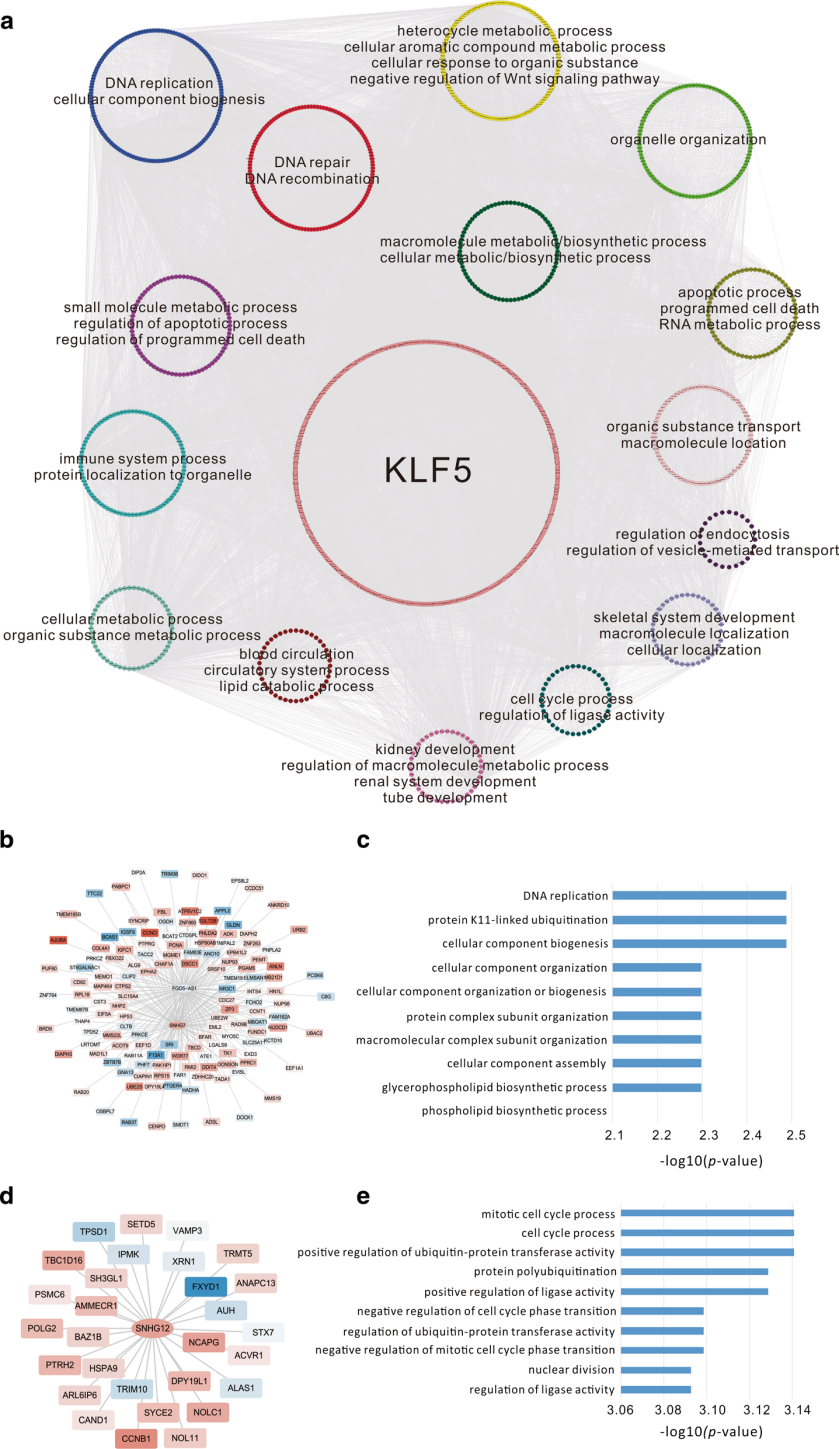
The visualization of the regulatory network within *KLF5* targets. **a** The 15 small circles are the 15 modules with gene numbers larger than 25 identified by the MCL algorithm, while the genes in the biggest circle are the other genes that did not belong to these modules. The main enriched GO BP terms are shown in each module. **b** The subnetwork visualization of the DNA replication module. The circle nodes represent PCGs while the triangle nodes represent lncRNAs. The red nodes represent the genes up-regulated in CRC while the green nodes represent the genes down-regulated in CRC. **c** The enriched GO BP terms of PCGs in the DNA replication module. **d** The subnetwork visualization of the cell cycle module. The meaning of the nodes with different colors are the same as (**b**). **e** The enriched GO BP terms of PCGs in the cell cycle module

The *KLF5* regulatory network was constructed based on the relationships between targets together with *KLF5*, PPIs among PCGs, and lncRNA-PCG co-expression. Finally, 14,823 lncRNA–protein co-expression relationships and 934 PPIs were obtained, with eight up-regulated and seven down-regulated lncRNAs, plus 927 up-regulated and 726 down-regulated PCGs (Fig. 3a). Among the genes in the network, *EP300* and *RARA* have previously been shown to be associated with *KLF5* in CRC [60, 61].

Next, we performed module analysis using the MCL algorithm with its default parameters [45], resulting in 15 modules with at least 25 genes. It is also important to note that the functional enrichment analysis showed that these modules were involved in DNA replication (module 1), DNA repair (module 2), stimulus-response (module 3), immune system process (module 5), apoptosis and cell death (module 6), metabolic and biosynthetic processes (modules 7, 8, and 10), development (module 11), and the cell cycle (module 14) (Fig. 3a). For example, module 1 included one up-regulated lncRNA, *SNHG7*, one down-regulated lncRNA, *FGD5-AS1*, and 138 PCGs, which has major roles in DNA replication and other biogenesis or biosynthetic process (Fig. 3b, c). On the other hand, module 14 included one up-regulated lncRNA, *SNHG1*2, and 37 PCGs, whose functions are mainly restricted to the cell cycle (Fig. 3d, e). The enriched functions of the modules suggest that *KLF5* and its targets may participate in similar biological processes.

### *SNHG12* in cell cycle module is up-regulated in CRC tissues and cell lines

**Fig. 4:**
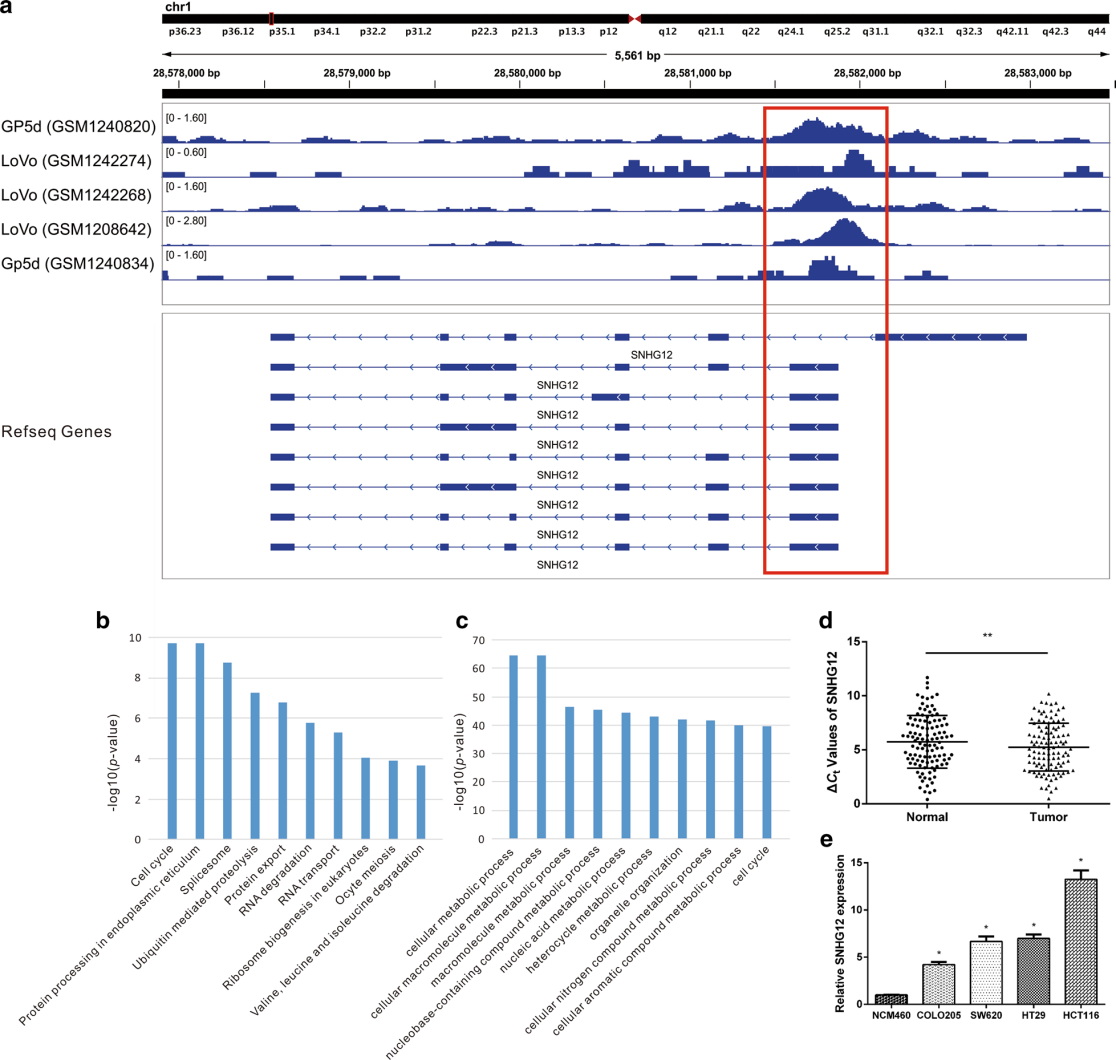
Peak visualization of 5 ChIP-Seq datasets of *KLF5* on *SNHG12* and its expression pattern. **a** The signals of the 5 ChIP-Seq datasets of *KLF5* are shown. The picture shows that the promoter of *SNHG12* has a high *KLF5* binding signal in 5 samples. **b** The top 10 enriched GO BP terms of *SNHG12* co-expressed partners in CRC. **c** The top 10 enriched KEGG pathways of *SNHG12* co-expressed partners in CRC. **d** The expression level of *SNHG12* in CRC tissues was significantly higher than that in adjacent normal tissues. **e** *SNHG12* expression levels were up-regulated in CRC cell lines (HCT116, HT29, SW620, and COLO-205) compared with the normal colon epithelial cell line (NCM460)

Considering the crucial role of *KLF5* in the cell cycle [11, 12], we focused on the module 14, with the enriched cell cycle function. *SNHG12*, the only lncRNA in this module, promotes tumorigenesis and metastasis in human osteosarcoma cells [34] and hepatocellular carcinoma [62]. In particular, we determined that there were *KLF5* binding sites in *SNHG12* promoter regions (Fig. 4a). Function prediction of *SNHG12* based on the co-expression network showed that *SNHG12* might be involved in metabolic processes and the cell cycle (Fig. 4b, c). For validation, we first examined the expression pattern of *SNHG12* in 111 matched CRC tissues and its corresponding adjacent normal tissues. The results showed that *SNHG12* (*p* < 0.01, Fig. 4d) was significantly up-regulated in CRC tissues. Next, the expression levels were evaluated in four CRC cell lines (HCT116, HT29, SW620, and COLO-205) and one human colon epithelial cell line (NCM460). After normalization to NCM460, a substantial induction of *SNHG12* was observed in all detected CRC cell lines (*p* < 0.05, Fig. 4e).

### *SNHG12* expression is regulated by *KLF5* in CRC cell line

**Fig. 5:**
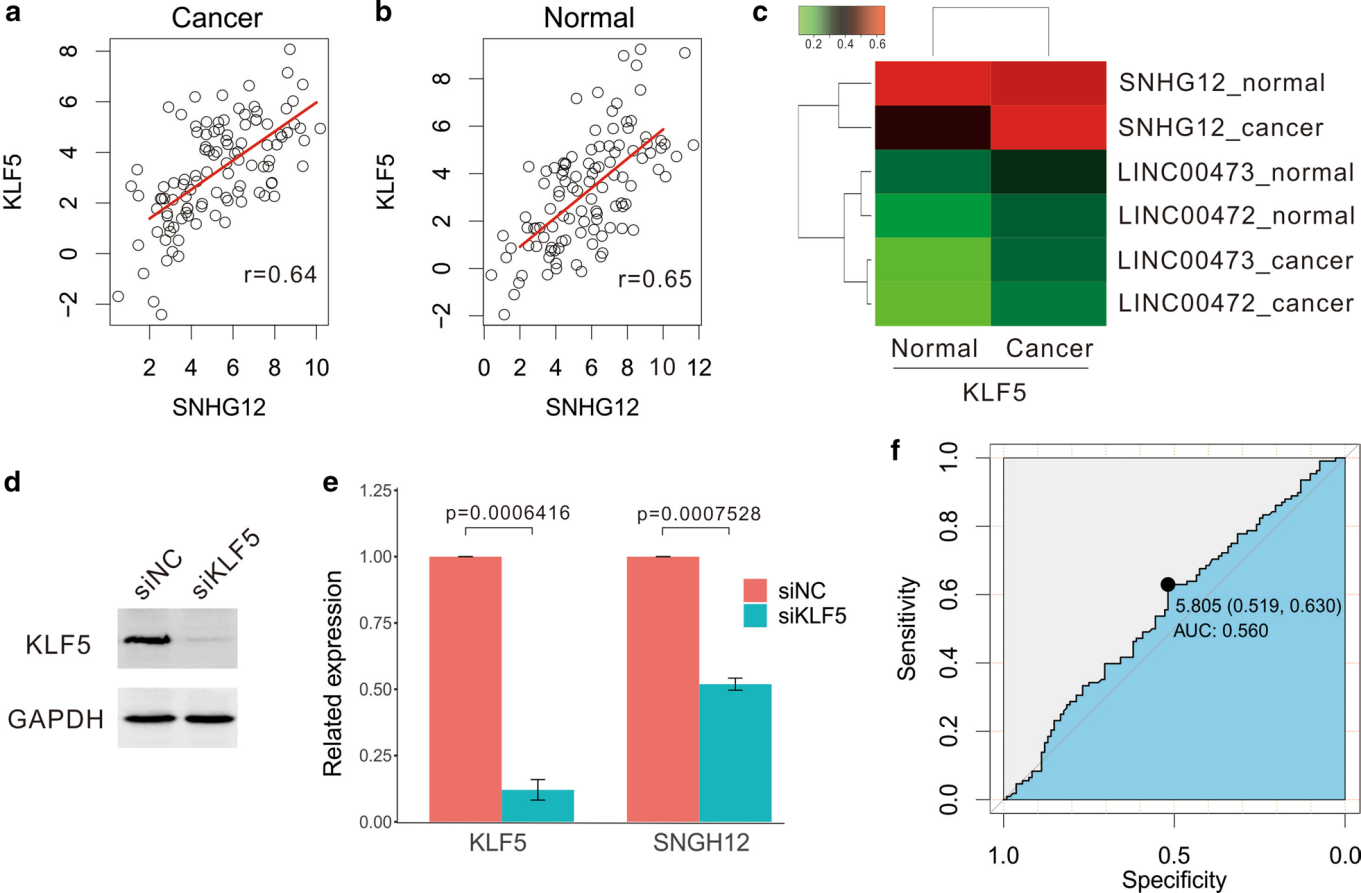
*SNHG12* is a potential target of *KLF5*. **a**, **b** The expression level of *KLF5* was positively correlated with *SNHG12* expression in both cancer (**a**) and normal (**b**) tissues. **c** The expression relationships between *KLF5* and *SNHG12* in cancer tissues and adjacent normal tissue were observed. Using the control, we showed the relationships between *KLF5* and *LINC00472*, *LINC00473*. **d** Comparison of *KLF5* protein expression in siNC and siKLF5 group by Western blot experiment. **e** Comparison of *KLF5* and *SNHG12* RNA expression in siNC and siKLF5 group by qRT-PCR experiment. **f** The ROC value of *SNHG12* is 0.56

To further determine the relationship between *SNHG12* and *KLF5*, we then studied the expression levels of *KLF5* in the same CRC tissue samples. As hypothesized, *KLF5* expression was positively correlated with that of *SNHG12* in both cancer and normal tissues (Fig. 5a, b). As a comparison, two other lncRNAs studied previously, *LINC00472* [63] and *LINC00473* (manuscript submitted), displayed a much lower PCCs (Fig. 5c). Furthermore, to verify the regulatory relationship of the two genes, biological experiments were further performed. First, Western blot experiment showed the success of knocking-down expression of *KLF5* in HCT116 cell line with siRNA (Fig. 5d). Second, using qRT-PCR, *SNHG12* expression was observed to be down-regulated significantly after knocking-down expression of *KLF5* with siRNA (Fig. 5e). Our results show *SNHG12* may be regulated by *KLF5*.

### Association between *SNHG12* expression and clinical-pathological characteristics of patients with CRC

To investigate the oncogenic role of *SNHG12* in CRC progression, we assessed the correlation between *SNHG12* expression levels and the clinical-pathological characteristics of CRC patients. As illustrated in Table 1, *SNHG12* expression was positively associated with invasion (*p* = 0.047) and distal metastasis (*p* = 0.03). However, we did not encounter any association between *SNHG12* expression and other clinical-pathological parameters such as age, gender, tumor location, tumor size, differentiation, lymph node metastasis, or TNM stage (Table 1).

**Table 1:** Association between *SNHG12* expression levels (Δ*C*_t_) and clinical-pathological factors

Characteristics	No. of patients (%)	*SNHG12* (Mean ± SD)	*p-*value
Age (year)			
≥ 60	67 (60.4)	5.25 ± 2.19	0.953
< 60	44 (39.6)	5.23 ± 2.27	
Gender			
Male	66 (59.5)	5.27 ± 1.97	0.886
Female	45 (40.5)	5.21 ± 2.54	
Tumor location			
Colon	54 (48.6)	4.95 ± 2.22	0.169
Rectal	57 (51.4)	5.53 ± 2.18	
Diameter (cm)			
> 5	28 (25.2)	5.12 ± 2.19	0.731
≤ 5	83 (74.8)	5.29 ± 2.23	
Differentiation			
Well	4 (3.6)	4.49 ± 2.04	0.692
Moderate	87 (78.4)	5.22 ± 2.25	
Poor	20 (18.0)	5.50 ± 2.11	
Invasion			
T1	7 (6.3)	6.79 ± 2.49	0.047*
T2	23 (20.7)	5.41 ± 2.24	
T3	16 (14.4)	5.91 ± 2.61	
T4	65 (58.6)	4.80 ± 1.92	
Lymph node metastasis			
N0	57 (51.4)	5.35 ± 2.25	0.253
N1	31 (27.9)	5.55 ± 2.26	
N2	23 (20.7)	4.58 ± 1.97	
Distal metastasis			
M 0	102 (91.9)	5.36 ± 2.20	0.030*
M 1	9 (8.1)	3.70 ± 1.68	
TNM stage			
I	21 (18.9)	5.56 ± 2.21	0.256
II	36 (32.4)	5.21 ± 2.30	
III	44 (39.6)	5.41 ± 2.18	
IV	10 (9.0)	3.96 ± 1.78	

A smaller Δ*C*_t_ value indicates higher expression

^*^ *p* values are significant under 0.05

Lastly, we reviewed whether *SNHG12* could be utilized as a diagnostic biomarker of CRC. Therefore, we constructed a Receiver Operating Characteristic (ROC) curves using adjacent normal tissues as a control, which resulted in an area of 0.56 under the ROC curve for *SNHG12* (Fig. 5f).

## Discussion

CRC is one of the most common malignancies, leading to high mortality rates worldwide [1]. The 5-year survival rate of patients with distant metastases is only 11% for colon cancer and 12% for rectal cancer [64]. Although some molecular markers, such as carcinoembryonic antigen, have been used for CRC diagnosis, their diagnostic values are not satisfactory, partially owing to different gene mutations and the complex protein–RNA regulatory network [2, 65]. In this study, a computational pipeline was designed to investigate the functional modules of *KLF5* in CRC and its downstream target lncRNA *SNHG12*, highlighting their oncogenic properties.

Recently, overwhelming evidence has indicated that any dysregulation in the expression of lncRNAs is associated with carcinogenesis and cancer metastasis [66]. As regulatory RNA molecules, lncRNAs have an essential role in the epigenetic, transcriptional, and post-transcriptional regulation of gene expression [67]. Additionally, many results have indicated that the abnormal expression of several lncRNAs is involved in the carcinogenesis of CRC [68, 69]. For instance, *HOTAIR* participates in CRC invasion and metastasis by stimulating chromatin modifications [22]. Taurine-upregulated gene 1 (*TUG1*), a lncRNA, functions as an oncogene in CRC by regulating cell proliferation, migration, and invasion [70]. Further experiments have demonstrated that *TUG1* knockdown could impair the migratory and invasive ability of CRC cells by regulating epithelial–mesenchymal transition [70]. Ge et al. [71] also found that prostate cancer-associated transcript 1 (*PCAT-1*) was up-regulated in CRC. Furthermore, research has shown that the overall survival of CRC patients with high *PCAT-1* expression was significantly lower than that of those with low expression [71]. *MEG3* expression was also significantly lower in CRC tissues compared to matched normal tissues, and *MEG3* down-regulation predicted poor prognosis in patients with CRC [72].

The biological network is a complex network that is involved in multiple types of molecular structures such as DNA, protein, and ncRNA. Acting as a TF, *KLF5* may co-operate with other TF to regulate targets together. In this study, we found *HSF1* is a potential co-factor of *KLF5* to regulate protein-coding genes. *HSF1* has been reported to have a central role in the heat shock response to maintain protein homeostasis in all eukaryotic cells [73]. In contrast, accumulating evidence revealed that *HSF1* has multiple additional functions, including roles in autophagy, apoptosis, immune response, cell growth arrest, and even cancer development [74]. The prominent role of *HSF1* has been observed in several cancers, including gastric cancer [75], osteosarcoma [76], breast cancer [77], and esophageal squamous cell carcinoma [78]. The up-regulation of *HSF1* promotes the proliferation, migration, and invasion of cancer cells and can serve as a prognostic marker in cancer [75]. In CRC, the expression of *HSF1* has been shown to be associated with metastasis, with respect to both RNA and protein levels [79, 80], indicating the key role of *HSF1* in CRC.

Regulatory ncRNAs such as miRNAs and lncRNAs have been shown to regulate the expression, stability, and subcellular location of PCGs in a various number of biological processes [81, 82]. Nowadays, several studies about the association between miRNAs and *KLF5* have been published [83–85]. For example, miR-5195-3p inhibits the proliferation and invasion of human bladder cancer cells by directly targeting *KLF5* [83]. Interestingly, *KLF5* acts as a transcription factor, which can regulate the expression of miRNA such as miR-200, to maintain epithelial characteristics and preventing epithelial-mesenchymal transition in epithelial cells [85]. Regarding lncRNAs, *KLF5* has been reported to regulate *LINC0346* in gastric cancer [86] and enhance the expression of *RP1* in breast cancer [87]. However, few studies were about lncRNAs and *KLF5* in CRC. The relationship between lncRNAs and TFs is mutual. On one side, lncRNAs can regulate TFs. On the other side, TFs can also regulate lncRNAs through the binding of its corresponding promoter regions just like PCGs.

In this research, we aimed to explore the PCG and lncRNA targets of *KLF5* and analyze the biological regulation network of *KLF5* in CRC. Through bioinformatics analysis, TFs *AR* and *HSF1* were found to be co-regulatory TFs with *KLF5* for lncRNAs and PCGs, respectively (Fig. 2a, b). Next, we showed that *SNHG12* function in the cell cycle module of *KLF5* may be vital in CRC (Fig. 3d, e). We verified the up-regulated expression patterns of *SNHG12* in CRC tissues and cell lines, and further examined the association with the clinical-pathological characteristics of patients with CRC (Fig. 4d, e, Table 1), proving that *SNHG12* is positively involved in CRC invasion and distal metastasis by having an oncogenic role in CRC. Furthermore, we observed *SNHG12* was down-regulated when expression of *KLF5* was knocked-down in CRC cell line (Fig. 5d, e), and the high correlation between *SNHG12* and *KLF5* expression in cancerous and normal tissues (Fig. 5a, b), suggesting that *SNHG12* is a potential target of *KLF5* in CRC. However, whether *KLF5* binding promoter of *SNHG12*, further biological experiments such as Chromatin immunoprecipitation (ChIP) should be conducted in feature.

## Conclusion

In conclusion, our results provided several potential PCG and lncRNA targets for *KLF5* in CRC and further demonstrated that one of the targets, *SNHG12*, might function as an oncogene in CRC. Whether targeting *KLF5*–*SNHG12* will produce any therapeutic benefits will require further investigation.

## Supplementary information

**Additional file 1: Figure S1.** The pipeline of identifying regulatory co-factors of *KLF5.*

**Additional file 2: Table S1.** Potential lncRNA targets of *KLF5* in CRC.

**Additional file 3: Table S2.** Potential protein-coding gene targets of *KLF5* in CRC.

## Data Availability

The data that support the findings of this study are available from the corresponding authors upon reasonable request.

## Abbreviations

*KLF5*: Kruppel like factor 5
PCGs: Protein coding genes
CRC: Colorectal cancer
lncRNAs: Long non-coding RNAs
*SNHG12*: Small nucleolar RNA host gene 12
LPA: Lysophosphatidic acid
*HOTAIR*: HOX transcript antisense RNA
GAS5: Growth arrest specific 5
*MEG3*: Maternally expressed gene 3
PPIs: Protein–protein interactions
CNC: Coding-non-coding gene co-expression network
HSR: Heat shock response
CEA: Carcinoembryonic antigen
*TUG1*: Taurine-upregulated gene 1
EMT: Epithelial–mesenchymal transition
*PCAT-1*: Prostate cancer-associated transcript 1
TCGA: The Cancer Genome Atlas
GO: Gene ontology
BP: Biological process
AJCC: American Joint Committee on Cancer
ANOVA: Analysis of variance
ROC: Receiver operating characteristic
