# Supplementary figures and images for: Network analysis of KLF5 targets showing the potential oncogenic role of SNHG12 in colorectal cancer
Source: Cancer Cell Int. 2020 Sep 7;20:439. doi: 10.1186/s12935-020-01527-x (PMC7487661; doi:10.1186/s12935-020-01527-x)

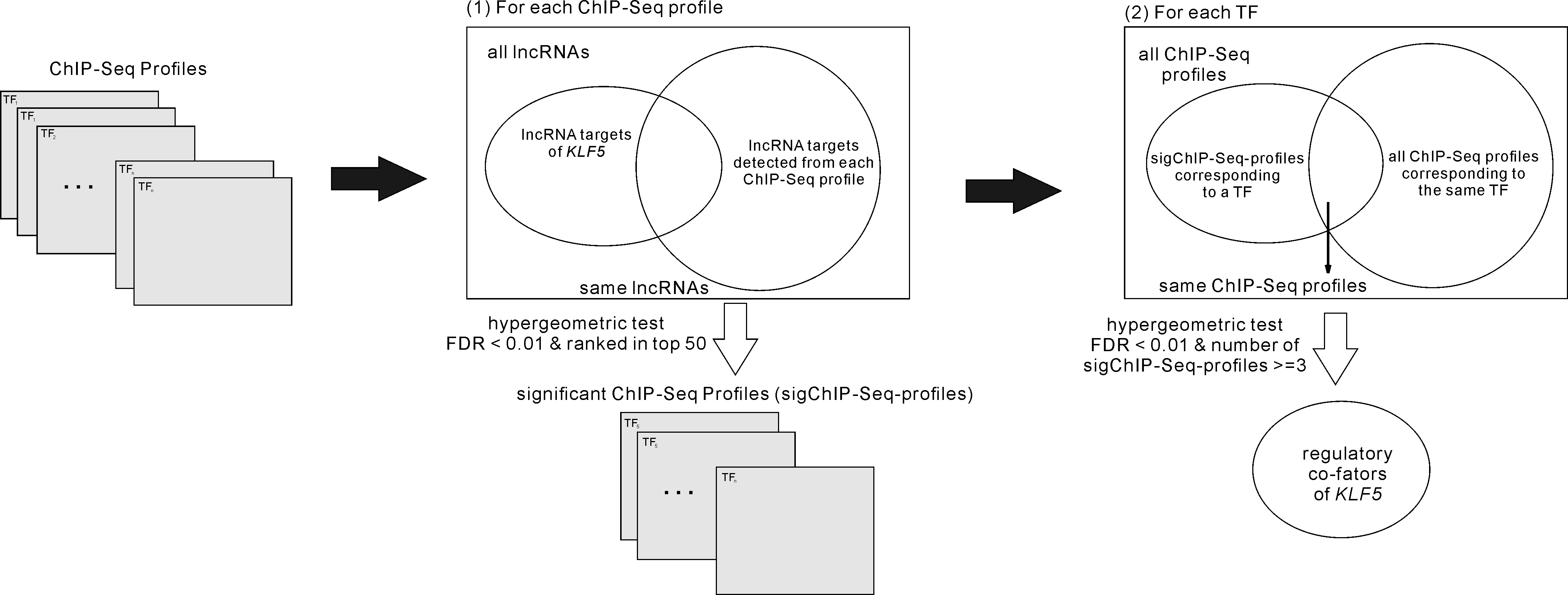

Supplement: Supplementary file 1 — Additional file 1: Figure S1. The pipeline of identifying regulatory co-factors of KLF5. [file 12935_2020_1527_MOESM1_ESM.jpg]
